# The Socioeconomic Indicators Linked to Parent Health-Related Technology Use: Cross-sectional Survey

**DOI:** 10.2196/37455

**Published:** 2022-11-30

**Authors:** Madison P McCall, Megan T Hineline, Margaret T Anton, April Highlander, Deborah J Jones

**Affiliations:** 1 Department of Psychology and Neuroscience University of North Carolina at Chapel Hill Chapel Hill, NC United States; 2 Department of Psychology Appalachian State University Boone, NC United States; 3 AbleTo, Inc New York City, NY United States

**Keywords:** parenting, child, health behavior, information seeking, health-related technology use, health information, digital health, mobile health, socioeconomic status, accessibility

## Abstract

**Background:**

Despite the prevalence of parent health information seeking on the internet and its impact on parenting behavior, there is a paucity of research on parents of young children (ages 3 to 8 years). Given the importance of this developmental period, exploring how family socioeconomic indicators linked to the digital divide and health inequities affect parent proxy- and self-seeking is critical to further understanding variability in health information seeking and associated outcomes.

**Objective:**

This study aimed to explore parental health-related technology use (HTU), the process by which parents engage in support, advice, and information-seeking behavior related to their (self-seeking) and their children’s (proxy seeking) health across a range of hardware devices (eg, tablet, wearable, smartphone, laptop, and desktop computer) and sources (eg, search engines, mobile applications, social media, and other digital media).

**Methods:**

A cross-sectional study including 313 parents and guardians of children ages 3 to 8 years recruited through Amazon Mechanical Turk (MTurk) was conducted. Parents were asked to complete a self-administered questionnaire on a broad range of parenting and parent-related constructs, including sociodemographic information, technology device ownership, and engagement in and use, features, and perceptions of HTU. Descriptive and bivariate analyses (chi-square tests) were performed to identify patterns and investigate associations between family socioeconomic indicators and parent HTU.

**Results:**

The overwhelming majority (301/313, 96%) of parents of young children reported engaging in HTU, of which 99% (300/301) reported using search engines (eg, Google), followed by social media (62%, 188/301), other forms of digital media (eg, podcasts; 145/301, 48%), and mobile applications (114/301, 38%). Parents who engaged in HTU reported seeking information about their child’s behavior and discipline practices (260/313, 83%), mental or physical health (181/313, 58%), and academic performance (142/313, 45%). Additionally, nearly half (134/313, 43%) of parents reported searching for advice on managing their stress. Among parents who reported using each source, an overwhelming majority (280/300, 93%) indicated that search engines were a helpful online source for proxy- and self-seeking, followed by social media (89%, 167/188), other digital media (120/145, 83%), and mobile apps (87/114, 76%). Among parents who reported using any technology source, approximately one-fifth reported that technology sources were most comfortable (61/311, 20%), most understanding (69/311, 22%), and most influential toward behavior change (73/312, 23%) compared to traditional sources of health information–seeking, including mental health professionals, other health care professionals, school professionals, community leaders, friends, and family members. Indicators of family socioeconomic status were differentially associated with frequency and perceptions of and search content associated with parent HTU across technology sources.

**Conclusions:**

The findings of this study underscore critical considerations in the design and dissemination of digital resources, programs, and interventions targeting parent and child health, especially for families in traditionally underserved communities.

## Introduction

In the past decade, researchers have shown an increased interest in parental online health information seeking (OHIS), the process by which parents search for health information using the internet, including search engines, forums, and social networking [1,2]. OHIS has been linked to various aspects of individual and family functioning, including parenting behavior, perceived social support, and health status [3-6]. While parents search for information related to their own health (ie, self-seeking), they are even more likely to use the internet for health information related to their children (ie, proxy seeking). Indeed, data from the past several years revealed that 75% to 90% of parents have searched for health information related to their child [1].

Despite the widespread prevalence of parent health information–seeking on the internet, there is a paucity of research among parents of young children ages 3 to 8 years [1]. Research indicates that up to one-third (15% to 30%) of young children experience social, emotional, and behavioral problems [6-9]. Further, difficulties during this critical developmental period can persist into adolescence and adulthood, increasing the risk for long-term academic, occupational, and physical and mental health difficulties [10,11], especially for children in traditionally underserved communities with less access to quality care [12]. Given the importance of early development in child and family health, exploring how sociodemographic characteristics linked to the digital divide and health inequities affect parent proxy- and self-seeking is critical to further understanding variability in health information–seeking behaviors in the community [13,14].

Accordingly, this study addresses 2 underdeveloped research areas with parents of young children. First, the bulk of work has focused on clinical or treatment-seeking samples of parents with specific presenting issues (eg, attention deficit hyperactivity disorder, hearing loss) or circumstances (eg, after childbirth, during a visit to a pediatric outpatient clinic). However, parents’ recognition of health-related concerns outside of the traditional health care system may depend on the extent to which they perceive a mismatch between their child’s functioning and the socially and culturally relevant contexts (eg, school, home) in which they engage in daily life. Further, such perceptions may prompt parents to search for health-related content within broader domains (eg, child academic performance, parental discipline) of child and family functioning. Considering the information-seeking behaviors of parents of young children experiencing chronic illnesses or acute health problems may not generalize to other parents, studies with non–treatment-seeking samples are critical to understanding health information needs, seeking behaviors, and outcomes across diverse families.

Second, prior studies investigating parent OHIS have been limited to internet use, defined broadly and inconsistently across studies [1,15]. Considering the increasing adoption and use of other consumer technologies (eg, mobile apps and wearables) for health-related reasons and long-standing disparities in broadband access and connectivity, there is a need to extend current work to account for parent use of a variety of information and communications technologies [16-19]. Accordingly, we refer to parental health-related technology use (HTU) as the process by which parents engage in support, advice, and information-seeking behavior related to their (self-seeking) and their children’s (proxy seeking) health across a broader range of devices (ie, tablets, wearables, smartphones, laptops, and desktop computers) and sources (ie, search engines, mobile applications, social media, and other digital media).

Building upon these gaps in the literature, this study aims to describe HTU among parents of young children, including the frequency and perceived usefulness of and search content associated with parent HTU in a non–treatment-seeking sample. In addition, resources (eg, parent access to technology devices) and perceptions (eg, comfortability) that may influence parent engagement in HTU are examined. Finally, whether patterns vary by parent, child, and household-level sociodemographic characteristics is explored.

## Methods

### Participant Recruitment

Parents and guardians of children ages 3 to 8 years old were recruited through Amazon Mechanical Turk (MTurk) to complete a survey on a broad range of parenting and parent-related constructs. Parents consented online before completing study measures in compliance with university-approved institutional review board (IRB) procedures. Upon confirming eligibility criteria, respondents were asked to select their youngest child in the specified age range to be referred to as the target child throughout the survey. All demographic variables and questionnaires were completed regarding the selected target child.

Additional measures were included to increase confidence in a participant pool that provides responses comparable to traditional samples (eg, [20-22]). To ensure attention to survey responses, 4 attention check questions were included throughout the survey (eg, “For data quality purposes, please select Sometimes”) and were assessed as part of the inclusion criteria. Additionally, respondents with duplicate IP addresses, geolocations, and MTurk IDs were excluded from analyses in accordance with recommendations for studies using MTurk samples. As with other crowdsourcing platforms, MTurk duplicates typically reflect multiple entries from the same individual or household or, most prominently, “bot” (ie, computer programs that can automatically complete surveys) or “farmer” respondents (ie, individuals using server farms or commercial data centers to evade MTurk’s screening procedures). Furthermore, these respondents are linked to lower-quality data [20]. Finally, a random numerical code was provided to eligible participants (ie, parents of children ages 3 to 8 years old living in the United States) upon completion of the study to facilitate participant payment of US $2.

### Ethics Approval

This study (17-0722) was approved by the institutional review board of the University of North Carolina at Chapel Hill.

### Measures

#### Sociodemographic Characteristics

Parents reported sociodemographic information for their family, including the age, race (eg, White, African American/Black, Asian or Pacific Islander, American Indian/Alaska Native, or multiracial), and ethnicity (eg, Hispanic/Latino) of both the respondent (ie, parent or caregiver) and target child. Multiple indicators of family socioeconomic status were also collected, including annual household income, parent employment status (eg, full-time employment, part-time employment, unemployed but looking for work, nonworking, and retired), parent educational attainment (eg, less than high school or General Education Diploma [GED], high school graduate or GED, some college, associate’s degree, bachelor's degree, master’s degree, and doctorate), and perceived financial difficulty. Finally, parents also reported their household composition, marital status, relationship to the target child, and the target child’s health status (ie, prior diagnosis of or treatment for developmental delays).

#### Technology Device Ownership, Access, and Use

Parents reported their access to and frequency of using common technology devices (ie, desktop computer, laptop computer, smartphone, tablet, wearable) measured on a 6-point Likert scale ranging from 0 (never) to 5 (more than once daily).

#### Search Content

Parents reported the content of their search for health-related information, advice, or support, focusing on 3 broad domains of proxy seeking (child academics, behavior, and mental and physical health) and 1 domain of self-seeking (parent stress and stress management).

#### Frequency and Usefulness of Parent Health-Related Technology Use

Parents indicated their use and perceptions of particular technology-enabled sources (eg, search engines, mobile apps, social media, and other forms of digital media) to search for parenting advice and health-related information for their children. Parents reported the frequency of using each source (ie, “When you are looking for parenting advice, information, and/or support, how often do you turn to each of the following potential sources?”) using a 4-point Likert scale ranging from 0 (never) to 3 (frequently). Although the usefulness of particular sources has been evaluated inconsistently in the literature on parent HTU (eg, [23,24]), researchers often use a single-item measure to capture the construct (eg, “How useful do you feel the internet is in helping you make decisions about your health?”) [25]. Similarly, parents reported the usefulness of a source (ie, “How helpful or useful did you find the parenting advice, information, and/or support you received from these sources?”) using a 4-point Likert scale ranging from 0 (not at all helpful) to 3 (very helpful).

### Statistical Analysis

Descriptive statistics were used to summarize family characteristics, parent ownership of or access to consumer technology devices, and parent engagement in and perceptions of HTU. Chi-square tests were conducted to compare proportions of and determine associations between device ownership and characteristics of HTU (eg, search content, frequency of use, and usefulness of technology sources) across groups defined by parent educational attainment (<bachelor’s degree vs ≥bachelor’s degree), perceived financial difficulty (none to mild vs moderate to severe), and low-income status, as determined by the federal poverty level (FPL), which accounts for annual household income and the number of people in the household (<200% FPL vs ≥200% FPL). Importantly, while “low-income” has been defined inconsistently in the literature, the FPL is typically used to determine eligibility for services, including those related to child health and development (eg, Head Start, Children’s Health Insurance Program) [26]. While income eligibility varies by state and service, 200% FPL has been mandated as an upper limit for participation in several government services (eg, Children’s Health Insurance Program, Subsidized Child Care Assistance Program), and incomes below 200% FPL account for a significant proportion of families in the United States who experience increased financial burden and economic insecurity [27]. Indeed, nearly 17% of children in the United States live in poverty, with approximately 7% (New Hampshire) to 56% (Puerto Rico) living in households below 200% FPL across the United States [28,29]. Of note, sociodemographic characteristics were included in analyses based on their theoretical relevance, as indicated in the previous research [13,18]. Missing values were excluded from analyses. Statistical analyses were conducted using SPSS version 26 software.

## Results

### Participants

Of the 657 respondents who completed the survey, 344 were removed from analyses for screening ineligibility (eg, families without a child in the specified age range or living outside of the United States, n=116), missed attention check questions (n=86), or duplicate IP addresses, geolocations, or MTurk IDs (n=142), yielding a total of 313 for analyses. Parents ranged in age from 19 to 57 years with a mean parental age of 34.19 (SD 7.11) years. Three-fifths (186/313, 59.4%) of parents self-identified as female. Slightly more than half (176/313, 56.2%) of parents obtained a bachelor’s degree or higher (ie, master’s or doctorate), and most were employed full- or part-time (280/313, 89.5%). Most parents were also married (243/313, 77.6%) and the biological parent of the target child (281/313, 89.8%). According to the parent report, approximately half (153/312, 49%) of the target children were female, and their mean age was 4.67 (SD 1.37) years. The racial and ethnic identity of most parents was White and non-Hispanic/Latino (230/312, 73.7%), followed by 8.7% (27/312) African American or Black, 4.5% (14/312) Asian American, 0.6% (2/312) American Indian/Alaska Native, and 3.2% (10/312) multiracial. For 10.5% (33/313) of the children, the parent’s self-reported race or ethnicity differed from that of the child. Nevertheless, the majority of children identified as White and non-Hispanic/Latino (207/312, 66.3%), followed by 7.4% (23/312) African American, 3.8% (12/312) Asian American, 0.6% (2/312) American Indian/Alaska Native, and 13.1% (41/312) multiracial. Additionally, 11.8% (37/313) of parents and 15.3% (48/313) of children identified as Hispanic or Latino. The annual combined household income ranged from US $6000 to $380,000 with a median of $60,000 (SD $41,180). Finally, 56.2% (176/313) of families reported living in suburban areas, followed by 25.2% (79/313) in urban areas and 18.5% (58/313) in rural areas. Compared to the general population of parents in the United States, the recruited sample included slightly more college-educated and lower-income participants and a comparable percentage of women and married parents [30,31]. Additionally, parents were less racially and ethnically diverse than the general population of parents in the United States but slightly more so than has been reported in previous studies with parents using MTurk samples.

### Device Ownership and Use

Parents reported owning a variety of technology devices, including a smartphone (276/313, 88.2%), laptop (276/313, 88.2%), tablet (243/313, 77.6%), desktop computer (193/313, 61.6%), and wearable device (100/313, 31.9%; Table 1). All (100%, 313/313) parents reported owning or having access to at least 1 technology device at home, and the majority (283/313, 90.4%) of parents reported access to multiple devices. Only 2 (0.64%) parents indicated not having access to a computer (desktop or laptop) at home, and both reported having access at work, school, or another setting (eg, library). Of the 37 (11.8%) participants that reported not having access to a smartphone, 12 (32.4%) reported having access to a smartphone at work, school, or another setting. Chi-square analyses revealed no statistically significant associations between parent educational attainment or perceived financial difficulty and access to technology devices. Families in low-income households were significantly less likely to own or have access to a wearable (*χ*^2^_1_=4.7, *P*=.03), but not any other technology device.

**Table 1:** Technology device ownership and access.

Demographics		Overall (N=313), n (%)	Desktop (n=193)			Laptop (n=276)				Smartphone (n=276)				Tablet (n=243)				Wearable (n=100)			
			Value, n (%)	*χ*^2^(*df*), *P* value		Value, n (%)		*χ*^2^(*df*), *P* value		Value, n (%)		*χ*^2^(*df*), *P* value		Value, n (%)		*χ*^2^(*df*), *P* value		Value, n (%)		*χ*^2^(*df*), *P* value	
**Parent educational attainment**					0.120 (*1*), .729				0.005 (*1*), .945				0.178 (*1*), .673				0.010 (*1*), .921				0.849 (*1*), .357
	<Bachelor’s degree	137 (43.8)	83 (60.6)			121 (88.3)				122 (89.1)				106 (77.4)				40 (29.2)			
	≥Bachelor’s degree	176 (56.2)	110 (62.5)			155 (88.1)				154 (87.5)				137 (77.8)				60 (34.1)			
**Household income**					0.246 (*1*), .620				2.676 (*1*), .102				0.147 (*1*), .701				0.075 (*1*), .785				4.744 (*1*), .034^a^
	<200% FPL^b^	95 (30.4)	57 (60.0)			88 (92.6)				85 (89.5)				73 (76.8)				22 (23.2)			
	≥200% FPL	216 (69.0)	136 (63.0)			186 (86.1)				190 (88.0)				169 (78.2)				77 (35.7)			
**Perceived financial difficulty**					0.028 (*1*), .868				0 (*1*), .998				0.591 (*1*), .442				0.127 (*1*), .721				1.563 (*1*), .211
	None to mild	220 (70.3)	135 (61.4)			194 (88.2)				196 (89.1)				172 (78.2)				75 (34.1)			
	Moderate to severe	93 (29.7)	58 (62.4)			82 (88.2)				80 (86.0)				71 (76.3)				25 (26.9)			

^a^*P*<.05.

^b^FPL: federal poverty level.

Among parents who reported access to a computer at home or another setting, 100% reported using their laptop or desktop device at least once monthly, with most reporting using their desktop computer (104/155, 67.1%) or laptop (117/177, 66.1%) more than once daily. Over half (31/55, 56.4%) of the parents reported using a wearable device multiple times during the day, and only 7% (4/55) reported using their wearable less than every 3 days. Approximately 31.1% (28/90) of parents who reported access to a tablet at home or another setting reported using their device more than once daily, and nearly a quarter (22/90, 24.4%) reported using their tablet once weekly or less. Most (177/196, 90.3%) parents reported using their smartphone multiple times per day, and no parents reported using their smartphone less than every 3 days.

The frequency of smartphone use was significantly lower for families in low-income households (*χ*^2^_2_=9.8, *P*=.007) and with parents reporting moderate to severe financial difficulty (*χ*^2^_2_=7.8, *P*=.021). Additionally, parents experiencing moderate to severe financial difficulty used their desktop computer (*Χ^2^_5_*=11.5, *P*=.042), laptop (*Χ^2^*_5_=12.4, *P*=.015), and tablet (*Χ^2^_5_*=23.9, *P*<.001) less frequently than their peers. The frequency of using any technology device did not vary significantly by parent educational attainment. Notably, parent age was not significantly correlated with the frequency of using any technology device.

### Parent HTU

Most parents (301/313, 96.2%) reported using technology sources to search for parenting advice and health-related information for their children. Parents who engaged in health-related technology use reported using search engines (eg, Google; 300/301, 99.7%), social media (188/301, 62.5%), other forms of digital media (eg, podcasts; 145/301, 48.2%), and mobile applications (114/301, 37.9%). Approximately one-third (91/301, 30.2%) of parents reported using all 4 sources for proxy- and self-seeking.

There were no significant differences between parents who did and did not report engaging in HTU via mobile apps, social media, and other digital media across parent educational attainment (Table 2). Parents in low-income households were significantly more likely to report using mobile apps (*χ*^2^_1_=4.7, *P*=.030) and social media (*Χ^2^*_1_=4.9, *P*=.026) for health-related reasons, but not other forms of digital media. Parents reporting moderate to severe financial difficulty were significantly more likely to report using mobile apps (*Χ^2^_1_*=5.5, *P=*.019), social media (*Χ^2^_1_*=4.2, *P=*.040), and other digital media (*Χ^2^_1_=*7.3, *P=*.007) in comparison to their peers. Given that most participants reported using search engines, the technology source was not included in the chi-square analyses.

**Figure 1:**
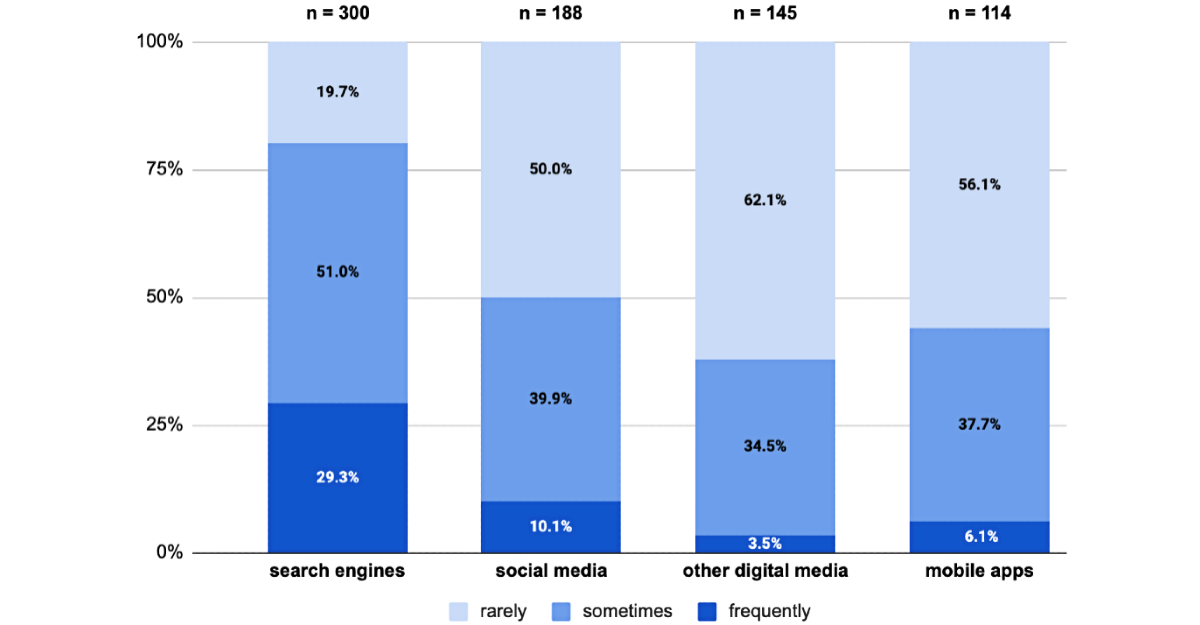
Frequency of parent health-related technology use (HTU) across technology sources.

While the frequency of engagement in HTU varied across sources, parents reported more frequent use of search engines on average, followed by social media, mobile apps, and other digital media (Figure 1). The frequency of parent use was not significantly associated with self-reported parent educational attainment. The mean frequency of social media use (*χ*^2^_3_=16.4, *P*<.001) was significantly greater for parents in low-income households, and the use of social media (*χ*^2^_3_=11.9, *P=*.008) and other digital media (*χ*^2^_3_=10.4, *P=*.016) was also increased for parents reporting moderate to severe financial difficulty.

Among parents who reported using each source, an overwhelming majority (280/300, 93.3%) indicated that search engines were a useful online source for proxy- and self-seeking, followed by social media (167/188, 88.8%), other digital media (120/145, 82.8%), and mobile apps (87/114, 76.3%). Parents in low-income households also rated other digital media as more useful than their peers (*χ*^2^_3_=9.19, *P=*.027). Perceived financial difficulty and parent educational attainment were not significantly associated with the perceived usefulness of any technology source.

**Table 2:** Parent engagement in health-related technology use (HTU).

Demographics		Overall (N=313), n (%)	Social media (n=188)					Other media (n=145)				Mobile apps (n=114)		
			Value, n (%)		*χ*^2^(*df*), *P* value		Value, n (%)		*χ*^2^(*df*), *P* value		Value, n (%)		*χ*^2^(*df*), *P* value	
**Parent educational attainment**						0.027 (*1*), .868				1.560 (*1*), .212				0.001 (*1*), .981
	<Bachelor’s degree	137 (43.8)	83 (60.6)				58 (42.3)				50 (36.5)			
	≥Bachelor’s degree	176 (56.2)	105 (59.7)				87 (49.4)				64 (36.4)			
**Household income**						4.983(*1*), .026^a^				0.982 (*1*), .322				4.714 (*1*), .030^a^
	<200% FPL^b^	95 (30.4)	66 (69.5)				48 (50.5)				43 (45.3)			
	≥200% FPL	216 (69.0)	121 (56.0)				96 (44.4)				70 (32.4)			
**Perceived financial difficulty**						4.226 (*1*), .040^a^				7.332 (*1*), .007^c^				5.504 (*1*), 0.019^a^
	None to mild	220 (70.3)	124 (56.4)				91 (41.4)				71 (32.3)			
	Moderate to severe	93 (29.7)	64 (68.8)				54 (58.1)				43 (46.2)			

^a^*P*<.05.

^b^FPL: federal poverty level.

^c^*P*<.01.

### Search Content

Parents who engaged in HTU reported seeking information about their child’s behavior and discipline practices (260/313, 83.1%), mental and physical health (181/313, 57.8%), and academic performance (142/313, 45.4%). Additionally, 42.8% (134/313) of parents reported searching for advice on managing their stress. Parents in low-income households were significantly less likely to search for health-related information or advice about their child’s physical and mental health (*χ*^2^_1_=5.0, *P=*.025) and more likely to search for content about parent stress and stress management (*χ*^2^_1_=12.2, *P*<.001; Table 3). Parents reporting moderate to severe financial difficulty were also more likely to search for the latter (*χ*^2^_1_=4.2, *P=*.041). Parent educational attainment was not significantly associated with any search content.

**Table 3:** Parent proxy and self-seeking content areas.

Demographics		Overall (N=313), n (%)	Child behavior/discipline (n=260)					Child academic performance (n=142)					Child physical/mental health (n=181)					Parent stress/stress management (n=134)		
			Value, n (%)		*χ*^2^(*df*), *P* value		Value, n (%)		*χ*^2^(*df*), *P* value		Value, n (%)			*χ*^2^(*df*), *P* value		Value, n (%)			*χ*^2^(*df*), *P* value	
**Parent educational attainment**						0.059 (*1*), .808				0.424 (*1*), .515					1.452(*1*), .228					3.695 (*1*), .055
	<Bachelor’s degree	137 (43.77)	113 (82.48)				65 (47.45)				74 (54.01)					67 (48.91)				
	≥Bachelor’s degree	176 (56.23)	147 (83.52)				77 (43.75)				107 (60.80)					67 (38.07)				
**Household income**						0.085 (*1*), .770				1.013 (*1*), .314					5.018 (*1*), .025^a^					12.231 (*1*), <.001^b^
	<200% FPL^c^	95 (30.35)	80 (84.21)				39 (41.05)				46 (48.42)					55 (57.89)				
	≥200% FPL	216 (69.01)	179 (82.87)				102 (47.22)				134 (62.04)					79 (36.57)				
**Perceived financial difficulty**						0.061 (*1*), .805				0.629 (*1*), .428					0.309 (*1*), .578					4.186 (*1*), .041^a^
	None to mild	220 (70.29)	182 (82.73)				103 (46.82)				125 (56.82)					86 (39.09)				
	Moderate to severe	93 (29.71)	78 (83.87)				39 (41.94)				56 (60.22)					48 (51.61)				

^a^*P<*.05

^b^*P<*.01

^c^FPL: federal poverty level.

### Perceptions

Among parents who reported using any technology source, approximately one-fifth reported that technology sources were the most comfortable (61/311, 19.6%), most understanding (69/311, 22.2%), and most influential toward behavior change (73/312, 23.4%) compared to traditional sources, including mental health professionals, other health care professionals, school professionals, community leaders, friends, and family members. For perceived understanding, the majority of parents (48/69, 69.6%) referenced search engines, followed by social media (19/69, 27.5%) and other digital media and mobile apps (both less than 1/69, 2%). Similarly, for perceived comfortability, most parents listed search engines (42/61, 68.9%) and social media (18/61, 29.5%), and fewer mentioned mobile apps (1/61, 1.6%) and other digital media (0/61, 0%). Finally, in terms of parenting behavior change, search engines accounted for 73.97% (54/73), followed by social media (16/73, 21.91%), other digital media (2/73, 2.74%), and mobile apps (1/73, 1.36%). Perceived financial difficulty, but not any other socioeconomic status (SES) indicator, was significantly associated with perceptions of technology sources for health information seeking, such that parents experiencing moderate to severe difficulty were more likely to perceive engagement in HTU as the most understanding (*χ*^2^_1_=14.2, *P*<.001), most comfortable (*χ*^2^_1_=7.9, *P=*.005), and most likely to lead to behavior change (*χ*^2^_1_=7.3, *P=*.007) compared to traditional sources (Table 4).

**Table 4:** Parent perceptions of health-related technology use (HTU).

Demographics		Overall (N=313), n (%)	Most understanding (n=69)					Most comfortable (n=61)				Most parenting behavior change (n=73)		
			Value, n (%)		*χ*^2^(*df*), *P* value		Value, n (%)		*χ*^2^(*df*), *P* value		Value, n (%)		*χ*^2^(*df*), *P* value	
**Parent educational attainment**						2.773 (*1*), .096				0.528 (*1*), .468				0.735 (*1*), .391
	<Bachelor’s degree	137 (43.8)	36 (26.7)				29 (21.5)				35 (25.7)			
	≥Bachelor’s degree	176 (56.2)	33 (18.8)				32 (18.2)				38 (21.6)			
**Household income**						0.978(*1*), .323				1.144 (*1*), .285				0.074 (*1*), .785
	<200% FPL^a^	95 (30.4)	24 (25.5)				22 (23.4)				23 (24.2)			
	≥200% FPL	216 (69.0)	44 (20.5)				39 (18.1)				49 (22.8)			
**Perceived financial difficulty**						14.169 (*1*), <.001^b^				7.851 (*1*), .005^b^				7.298 (*1*), .007^b^
	None to mild	220 (70.3)	36 (16.4)				34 (15.5)				42 (19.2)			
	Moderate to severe	93 (29.7)	33 (35.9)				27 (29.3)				31 (33.3)			

^a^FPL

^b^*P*<.01

## Discussion

### Principal Findings

Given the increased prevalence of parent health-related technology use in recent years, this study aimed to explore family socioeconomic factors associated with this parenting behavior in a diverse sample of parents of young children. Considering that several developmental, socioemotional, and behavioral problems emerge in early childhood, understanding parent HTU use during this period has numerous clinical and public health implications. Indeed, children from lower SES households are more likely to experience reduced health quality and are less likely to have access to traditional health care services than children from higher SES households, and the relationship between these disparities and long-standing structural barriers is well established [32]. Further, research suggests similar barriers persist in access to technology devices and broadband, which may also challenge recent efforts to leverage technology to address health disparities [17,33,34]. Thus, understanding patterns and perceptions of parental HTU is critical for efforts to democratize digital health for parents of young children.

In the past decade, there has been a significant increase in technology device ownership in the United States, most substantially among smartphones and tablets [19]. Recruited families displayed a slightly higher percentage of smartphone, computer, tablet, and wearable device ownership and access in comparison to recent surveys of US adults [16,19], which may be reflective of our focus on parents (rather than adults in general), recruitment methods (eg, telephone interviews vs Amazon Mechanical Turk), or the inclusion of families in analyses with access to devices in other settings (eg, work, school, or library). Over three-fifths of parents endorsed ownership or access to a smartphone, tablet, and desktop or laptop computers, which did not vary across educational attainment, perceived financial difficulty, or household income. However, fewer than a third of parents reported access to a wearable device, and families with a lower income were significantly less likely to own a wearable (23% vs 36%).

Importantly, the majority of parents of young children reported using their laptop (150/177, 85%) or desktop computer (130/155, 84%) and wearable devices (45/55, 82%) daily, and the overwhelming majority of parents reported using their smartphone more than once per day. In contrast, only half of the parents reported using their tablet daily. Some, but not all, indicators of SES were significantly associated with how often parents used their smartphone (income and perceived financial difficulty), tablet (perceived financial difficulty), and desktop computer (parent educational attainment), with parents without a bachelor’s degree, those experiencing moderate to severe financial difficulty, and those in lower-income households using their technology devices less frequently than their peers.

Regarding engagement in HTU among parents of young children, our findings were congruent with the high rates observed in previous studies of parent health information seeking via the internet [1]. However, these results extended the existing research by examining differential engagement across technology sources (eg, search engines, mobile apps, social media, and other digital media) in general and across sociodemographic groups. Consistent with previous research, nearly all parents in our study endorsed the use of search engines. In general, fewer parents reported using social media for health-related reasons in comparison to estimates of general social media use by parents (62% v 75%) [35]; however, existing work has primarily examined parents of infants, toddlers, or children under 18 years of age broadly [15]. Findings also indicate that less than half the parents of young children currently use mobile apps (38%) and other digital media (48%) to search for health-related information, advice, or support.

Additionally, there have been inconsistent findings regarding the relationship between family SES and parent HTU. For objective dimensions of SES, this is partly attributable to the underreporting of household income, household composition, and parent educational attainment in studies (40% did not report the education level of participants in a recent meta-analysis), as well as the recruitment of predominantly highly educated (over 50% to 75% with an academic degree) and higher-income parents among remaining studies [1]. Furthermore, to our knowledge, no studies to date have included subjective dimensions of SES in analyses (eg, perceived financial hardship, subjective social status), despite their distinct effects on parenting behavior and family health [36-41]. In contrast to studies observing higher rates of health-seeking behavior via the internet with increased parent educational attainment [1,42,43], our findings suggest no significant associations between parent educational attainment and engagement in or frequency of health-related technology use across sources. However, parents in lower-income households and those experiencing greater financial difficulty were significantly more likely to use social media (69% vs 56% for both) and mobile apps (45% vs 32% and 46% vs 32%, respectively) for health-related reasons. Parents who reported greater financial difficulty were also more likely to use other forms of digital media (58% vs 41%). Moreover, parents experiencing moderate to severe financial difficulty used social media less frequently than their peers. In terms of search content, both lower income and increased perceived financial difficulty (52% vs 39%) were associated with increased self-seeking behavior related to parent stress and stress management, and lower income was additionally associated with a decreased likelihood of parent engagement in proxy seeking related to their child’s mental and physical health. Finally, parent perceptions of health-related technology use broadly did not vary by any objective dimensions of SES; however, parents experiencing moderate to severe financial difficulty were significantly more likely to perceive technology sources as the most comfortable (29% vs 15%), understanding (36% vs 16%), and likely to influence behavior change (33% vs 19%) compared to traditional sources. These findings support early research suggesting that SES indicators have differential impacts on health behavior and outcomes, providing a basis for further exploration of the underlying mechanisms contributing to outcomes in parent HTU.

Taken together, the results of this study underscore potential considerations for clinicians, researchers, and public health practitioners engaged in the design and dissemination of digital resources, programs, and interventions targeting family health and well-being. For instance, our findings suggest that digital health tools developed with greater attention to the types of technology sources parents prefer for health-related information, their frequency of engagement with these sources (eg, daily or weekly), and the availability of technology devices required to access these sources may yield increased uptake. Further, our results suggest practical considerations for efforts striving to optimize effectiveness (eg, which commercial devices and sources have the necessary features and functionality?), scalability (eg, what are the current estimates of, trends in, and barriers to adoption of these devices, especially in historically excluded communities?), and sustainability (eg, how acceptable and usable are both the devices and sources for the target population?). For example, digital resources, programs, and interventions requiring devices compatible solely with mobile operating systems (eg, mobile apps for Android, Apple iOS, and iPadOS) may call for a consideration of parent access to, familiarity with, and perceptions of smartphones and tablets, as well as their perceptions of mobile apps as a source for health-related information and support. Importantly, the success of these efforts hinges on broader attention to policies that address the structural information, infrastructure, and implementation barriers to diverse parents’ safe and effective engagement in HTU, such as access to technology devices and reliable internet (eg, [44]) and threats to online safety (eg, health misinformation and disinformation [45-47]).

### Limitations and Future Directions

Despite its strengths, this study has some limitations. First, primarily descriptive analyses were conducted to explore associations between sociodemographic factors and outcome variables. Second, inadequate representation of all racial and ethnic groups precluded our ability to examine how the diversity of social context and experiences across and within groups influence health-related technology use, which is a critical step in future research given the well-established disparities in digital adoption, health outcomes, and access to care among racially and ethnically minoritized children and their families [48-51]. Third, sources were grouped into technology (ie, search engines, mobile apps, social media, and other digital media) and traditional (ie, family, friends, mental health care providers, other health care providers, school professionals, and community leaders) categories for the analyses of perceptions of HTU, despite their potential interconnections in daily life (eg, use of social media to connect with family members about child-related health concerns, use of telemedicine apps for remote health care services). Future research should explore these complex relationships, which are likely linked to other relevant individual (eg, parent and child psychosocial factors and attitudes) and environmental (eg, social support, discrimination) factors associated with engagement in HTU and outcomes (eg, specific parent behaviors, family health outcomes, subsequent HTU). Finally, survey data were collected in late 2018 (prior to the COVID-19 pandemic), spotlighting the importance of future work examining potentially evolving trends in technology adoption and parent HTU.

### Conclusion

In summary, this study investigated engagement in support, advice, and information-seeking behavior among parents of young children across technology devices and sources. It also examined resource access and perceptions that may influence engagement and explored patterns across family SES. Overall, this study supports the growing body of evidence demonstrating the potential for digital technologies to disseminate health-related information, support, and resources to young children and families facing structural socioeconomic barriers. Furthermore, it may inform future research necessary to advance understanding on how to more optimally tailor and deliver supports that benefit the health and well-being of all children.

## Abbreviations

FPL: federal poverty level
GED: General Education Diploma
HTU: health-related technology use
IRB: institutional review board
MTurk: Amazon Mechanical Turk
OHIS: online health information seeking
SES: socioeconomic status
